# Vulcanite and Celluloid

**Published:** 1884-05

**Authors:** 


VULCANITE AND CELLULOID.—Instructions in their Practical Working for Dental Purposes. By L. Eldred Gilbert, D. D. S., Instructor of Clinical Dentistry in the Philadelphia Dental College. With illustrations. Publishers: The S. S. White Dental Manufacturing Co. Price, cloth, 75 cents.

This is a concise monograph which will prove of great service to the dental student inasmuch as its author confines his descriptions of the methods of manipulating vulcanite and celluloid to a few reliable processes. Easily comprehended directions are given for each step of the process, from the taking of the impression to the completion of the denture; and the very moderate price of this useful little work, will enable every dental student to add it to the number of his text books. It will certainly be appreciated by every practitioner of dentistry who may peruse it, as it contains a great amount of useful information.

